# Genomic prediction of optimal cross combinations to accelerate genetic improvement of soybean (*Glycine max*)

**DOI:** 10.3389/fpls.2023.1171135

**Published:** 2023-05-10

**Authors:** Mark J. Miller, Qijian Song, Benjamin Fallen, Zenglu Li

**Affiliations:** ^1^ Institute of Plant Breeding, Genetics and Genomics, and Department of Crop and Soil Sciences, University of Georgia, Athens, GA, United States; ^2^ Soybean Genomics and Improvement Laboratory, United States Department of Agriculture - Agricultural Research Service, Beltsville, MD, United States; ^3^ Soybean and Nitrogen Fixation Research Unit, United States Department of Agriculture - Agricultural Research Service, Raleigh, NC, United States

**Keywords:** cross combination, molecular markers, genomic selection, cross prediction, soybean breeding

## Abstract

Improving yield is a primary soybean breeding goal, as yield is the main determinant of soybean’s profitability. Within the breeding process, selection of cross combinations is one of most important elements. Cross prediction will assist soybean breeders in identifying the best cross combinations among parental genotypes prior to crossing, increasing genetic gain and breeding efficiency. In this study optimal cross selection methods were created and applied in soybean and validated using historical data from the University of Georgia soybean breeding program, under multiple training set compositions and marker densities utilizing multiple genomic selection models for marker evaluation. Plant materials consisted of 702 advanced breeding lines evaluated in multiple environments and genotyped using SoySNP6k BeadChips. An additional marker set, the SoySNP3k marker set, was tested in this study as well. Optimal cross selection methods were used to predict the yield of 42 previously made crosses and compared to the performance of the cross’s offspring in replicated field trials. The best prediction accuracy was obtained when using Extended Genomic BLUP with the SoySNP6k marker set, consisting of 3,762 polymorphic markers, with an accuracy of 0.56 with a training set maximally related to the crosses predicted and 0.4 in a training set with minimized relatedness to predicted crosses. Prediction accuracy was most significantly impacted by training set relatedness to the predicted crosses, marker density, and the genomic model used to predict marker effects. The usefulness criterion selected had an impact on prediction accuracy within training sets with low relatedness to the crosses predicted. Optimal cross prediction provides a useful method that assists plant breeders in selecting crosses in soybean breeding.

## 1 Introduction

Parental selection and determination of cross combinations are initial, essential steps in soybean breeding. Most often, these crossing decisions are made on the basis of pedigrees, genetic diversity, yield, and agronomic traits (Bernardo, 2003; Gaynor et al., 2017). The following breeding stage, population development, lasts for several years after which agronomically important traits, such as yield, can be evaluated to determine if a crossing decision will result in a new cultivar (Heffner et al., 2010). A poor cross combination will therefore use up resources for multiple years prior to yield evaluation without resulting in useful cultivars. Furthermore, for a given set of parental lines, a breeder can only perform a small subset of all cross combinations possible. Assuming a panel of 100 parents, there are 4,950 unique cross combinations, more than can be made by any breeding program. A predictive method which would allow breeders to assess a cross at the beginning of the breeding cycle would alleviate both these issues with parental and cross combination selection, leading to better genetic gains and more efficient breeding methodologies.

Predictive, genomics-based breeding techniques, notably genomic selection, have been a major focus of study in both animal and plant breeding (Meuwissen et al., 2001; Bernardo and Yu, 2007; Habier et al., 2010; Crossa et al., 2014; Lorenz and Smith, 2015; Garcia-Ruiz et al., 2016; Crain et al., 2018; Stewart-Brown et al., 2019). These techniques utilize genomic and phenotypic datasets to predict the value of an individual based on their genotypic data, enabling selection in earlier generations without extensive field testing (Bernardo and Yu, 2007; Heffner et al., 2010; Cui et al., 2020). Additionally, genomic selection can reduce breeding cycle time by replacing preliminary replicated trials, leading to an increased rate of genetic gain (Schaeffer, 2006; Heffner et al., 2010; Rajsic et al., 2016; Gaynor et al., 2017; Gorjanc et al., 2018). This shortening of breeding cycles is most dramatic in breeding programs with longer breeding cycles, such as perennial crops or animal breeding (Schaeffer, 2006; Garcia-Ruiz et al., 2016; Lin et al., 2017). Genomic selection requires uniform genotypes for reliable predictions, therefore it can only be used following population development (Voss-Fels et al., 2019). In current plant breeding methodologies, the use of genomic selection differs for inbred line and hybrid development. For inbred species, primary purpose of genomic selections is used for *per se* evaluation, while it is used for parental line selection in hybrid development (Beyene et al., 2019; Stewart-Brown et al., 2019; Islam et al., 2020; Atanda et al., 2021).

Prediction of cross performance has been utilized for hybrid production in allogamous crops. The success of F_1_ hybrids is a function of each hybrid parents’ general combining ability and the specific combining ability of the cross. These characteristics require multiple rounds of testcrossing to accurately determine, making such tests a central part of the hybrid production cycle (Zhao et al., 2015; Cui et al., 2020). To aid in the development process, multiple hybrid prediction models have been proposed and tested for hybrid development (Marulanda et al., 2016; Beyene et al., 2019). These models allow for prediction of hybrid performance without field trials, based on the genetic data from the parents and their past performance in testcrosses. This allows breeding programs to bypass early rounds of testcrossing, accelerating the hybrid development process (Marulanda et al., 2016; Beyene et al., 2019; Cui et al., 2020).

The hybrid prediction methods proposed by Marulanda et al. (2016) and Longin et al. (2015), utilized genomic selection to evaluate double haploids for general combining ability in wheat, maize, and rice. After genomic selection, further testcrosses are utilized to make advancement decisions. Marulanda et al. (2016) found the greatest genetic gain when applying genomic selection once, following nursery selection for highly heritable traits. Genomic selection was then followed by one stage of phenotypic selection *via* testcrossing before release to registration trials. This increased genetic gain over traditional phenotypic selection was maintained as long as prediction accuracy was above 0.2 (Marulanda et al., 2016). These results were similar to those of Longin et al. (2015) and Beyene et al. (2019) who validated this pipeline in wheat and tropical maize breeding programs, respectively.

As a parallel to hybrid prediction, cross prediction methods for varietal development allow breeders to predict the value of the recombinant inbred lines (RILs) that a cross will generate, though there are key differences between predicting crosses for hybrids and inbred varieties. First, marker effects must be assessed across all RILs within the breeding population rather than on a per population or heterotic group basis, as is done in hybrid genomic selection (Schrag et al., 2009; Zhao et al., 2015). Additionally, the progeny genotypes of a cross for an inbred species are not uniform among full siblings as they are in hybrids. This means that a cross cannot be valued on the basis of uniform progeny with identical genetics, invalidating many of the assumptions of hybrid prediction. Instead, a given cross will generate a multitude of progeny genotypes which need to be statistically evaluated to determine the value of said cross.

Prediction of cross values for inbred varieties requires the prediction of progeny genotypes in order to predict progeny genetic values and performance. Bernardo (2014) investigated the concept of locus classification, in which loci were classified as beneficial or detrimental for a specific mating combination. It was theorized that more genetic gain would occur by crossing a parental line with plant materials that had complementary alleles, rather than crossing to a high performing, but genetically similar inbred. The genetically similar inbred and parental line would likely share many of the same detrimental alleles. By contrast, if a large or infinite number of progeny were created from a complementary mating, in one of the progeny the positive alleles of one parent would completely replace the negative alleles of the other parent and vice versa. This would lead to an optimal progeny with the greatest possible genetic gain. Optimal cross selection (OCS) advanced this concept, identifying complementary mating pairs *via* estimation of population mean and genetic variance in inbred species such as barley and wheat (Mohammadi et al., 2015; Lado et al., 2017). Mohammadi et al. (2015) is particularly notable among these studies, having created the PopVar package in R, which can perform OCS using ridge regression BLUP (RR-BLUP) to assess marker effects, and validated it using elite barley breeding lines. Within these OCS methods, the value of a cross is determined by a modified usefulness criterion (UC). The original form of this measurement was proposed by Schnell and Utz (1976) to estimate the genetic variability present in F_1_ progeny. The UC used in current GS studies modifies the equation by removing heritability as a factor, as was originally suggested by Zhong and Jannink (2007). With this modification, the UC represents the genetic gain possible within a bi-parental population (Zhong and Jannink, 2007; Bernardo, 2014; Mohammadi et al., 2015; Neyhart and Smith, 2019). The prediction methods used in prior OCS studies focused on high heritability traits well suited to prediction. Therefore, the models chosen in these studies to evaluate marker effects may not be appropriate for the agronomic traits with lower heritability, such as yield (Bernardo, 2014; Mohammadi et al., 2015; Wang et al., 2018; Stewart-Brown et al., 2019).

The objectives of this study were to evaluate multiple existing and novel models for yield prediction in cross combinations of soybean, validate them using historical breeding data under a multitude of conditions and parameters, and assess their uses in breeding programs. These parameters include the genomic evaluation method used for progeny analysis, the relatedness of the training set (TS) to parental lines, the UC used to determine cross value, as well as the marker density.

## 2 Materials and methods

### 2.1 Plant materials

The TS used in this study consisted of 702 elite, inbred soybean lines from the University of Georgia (UGA) Soybean Breeding Program’s advanced yield trials (AYTs). Thirty-five of these lines were used as parents in crosses made between 2012 and 2014. These parental lines were selected to be included in this study due to the availability of seed and their parentage in crosses which generated more than 15 progeny lines that were tested in replicated field trials (**Table S1**). Materials in the TS ranged from maturity groups (MGs) VI through VIII. These materials had been used to develop cultivars for commercial release in the Southern United States (Boerma et al., 2012; Boerma et al., 2016; King et al., 2016; Li et al., 2020).

Yield trials were conducted at three locations with three replicates in each location: Athens, Plains, and Tifton in Georgia and Florence, South Carolina. Plots consisted of four rows with a row length of 4.9 m and 76.2 cm row spacing, and a planting density of 27 seeds/meter. All plots were end-trimmed to a row length of 3.7 m the before harvest with only the middle two rows harvested for yield to reduce edge effects. Each trial followed a randomized complete block design and included a set of two checks with similar maturity. The 35 parental lines were primarily evaluated during 2007 to 2013 growing seasons, with all other lines evaluated between 2015 and 2019.

Additional plant materials were also included to calculate the value of the validation crosses drawn from the UGA Soybean Breeding Program’s preliminary yield trials (PYTs). For each of the 42 crosses utilized in validation, progeny from both the UGA Soybean Breeding Program’s AYT and PYT were used to calculate the observed value of the cross. PYTs were conducted similar to the AYTs with the exception that plots consisted of only two rows and were tested at two locations with two replicates in each location. These plant materials from the PYT were not included in the TS or used to predict cross values.

### 2.2 Genotyping

DNA was extracted from bulked, lyophilized leaf tissue collected from ~15 plants of each genotype. Extraction was accomplished using a modified CTAB, chloroform method detailed in Keim (1988). DNA quality and quantity were randomly checked *via* gel electrophoresis and fluorimeter DNA quantification with a minimum desirable concentration of 100 µg/mL for genotyping.

Genotyping was accomplished using the SoySNP6k iSelect BeadChip. This genotyping method was developed based on the analysis of the linkage disequilibrium and minor allele frequency using >18,000 accessions in the USDA Soybean Germplasm Collection. In validation using a diverse set of germplasm, the chip had a minor allele frequency of >0.1 for >90% of the markers included in the chip (Song et al., 2020). Following genotyping, SNP allele calls were manually checked for quality control using GenomeStudio (Illumina, San Diego, CA) with corrections made when necessary. SNP Markers with a minor allele frequency of<0.06 and/or >20% missing data were excluded from analysis, leaving 3,762 polymorphic markers for further analysis. The SNP markers were coded as 2, 1, or 0, corresponding to the number of alleles from the nucleotide variant first in alphabetical order for said marker. Missing SNP marker alleles were imputed using the software Beagle (version 5.0) (Browning et al., 2018). Additionally, a marker set of reduced density which consisted of markers included in the SoySNP3k iSelect BeadChip was tested (unpublished data). The 3k SNP marker set is a subset of markers drawn from the 6k SNP marker set and was filtered under the same parameters for minor allele frequency and missing data, which resulted in 2,020 remaining polymorphic SNP markers for further analysis. Minimal heterozygosity was found in the plant materials as all materials included in this study were RILs. Both marker sets are available to the soybean community.

### 2.3 Phenotypic data analysis

Grain yield at each location was collected from the middle two rows within each four-row plot after end-trimming. Yield was calculated based on the weight gathered from the combines during harvest of the yield plots, with yield adjusted to a standardized moisture of 13%. Maturity was evaluated continuously and collected as the days from planting to R8 when maturity was achieved by 95% of plants within a plot.

Best Linear Unbiased Prediction (BLUP) values for each line were calculated *via* a mixed model, which accounted for environmental, maturity, and genotype effects along with genotype by environment interactions. Environment was defined as a combination of year and location. Genotypes and genotype by environment interactions were treated as a random effect and environmental and maturity effects were treated as fixed effects in BLUP calculation. Histograms and residual plots were used to check for outliers in the raw phenotypic data, as well as the BLUP values, with flagrant outliers excluded from the analysis.

The broad-sense heritability (*H^2^*) was calculated for each yield trial in the TS utilizing data from the two testing locations where maturity data was available. Variance components were determined using a linear model equation in the form of:


y=μ+E+M+E(r)+G+E(G)+e

Where y denotes the yield, M is maturity measured in number of days from planting, E is the environmental effect, E(r) is the effect of the replication in a given environment, G is the genotypic effect, E(G) is the genotype by environment interaction, and *e* is the error term. G and E(G) were treated as random effects, while M, E, and E(r) were treated as fixed effects. Calculation was performed using the lme4 package in R (Bates et al., 2015). *H^2^* was calculated on an entry mean basis for each test using the following equation:


$$H^{2}=\sigma_{\;G}^{2}/\left(\sigma_{\;G}^{2}+\frac{\sigma_{\;GxE}^{2}}{e}+\frac{\sigma_{\;R}^{2}}{er}\right)$$

Where $\sigma_{\;G}^{2}$ , $\sigma_{\;GxE}^{2}$ , and $\sigma_{\;R}^{2}$ are the variance of genotypes, the genotype by environment interactions, and residuals, respectively; *e* is the number of environments for the test and *r* is the number of replications.

### 2.4 Optimal cross selection

#### 2.4.1 Progeny generation

Progeny were generated using parental genotypes *via* a simulation of the single seed descent method. Each parent consisted of 3,762 SNPs coded as 2, 1, or 0, corresponding to the number of alleles from the nucleotide variant first in alphabetical order for said marker. SNPs coded as 2 and 0 are therefore homozygous, with heterozygotes coded as 1. For each cross, 500 progeny were generated using the sim.cross function of the R/qtl package (Broman et al., 2003) in R, utilizing the genetic map associated with the SoySNP6k iSelect Beadchip (Mohammadi et al., 2015; Song et al., 2020). The progeny generation process assumed that all progeny were developed *via* a single seed descent method, with each of the progeny generated from a separate meiotic event within the F_1_ hybrids. Each resultant F_2_ genotype was then advanced through multiple generations of inbreeding to generate an F_5_ RIL. Meiotic events operated under the chi-squared model of crossover interference, with parameters similar to those of the PopVar package, assuming no crossover interference (Broman et al., 2003). R/qtl generated a generic RIL population for each cross, in which the alleles from each parent were coded as A or B and were replaced with the specific nucleotides of each parent for that marker. Genotypes generated were assumed to be F_5_-derived RILs. This process was carried out separately when utilizing the 3k SNP marker set. Within the UGA soybean breeding program, typically, 100 to 300 progeny rows were grown from each cross for evaluation. Therefore, to simulate the number of plant rows present during evaluation, 500 progeny genotypes were generated for each cross combination.

#### 2.4.2 Genotypic prediction & cross analysis

Following progeny generation, each cross combination was evaluated on the basis of the GEBVs predicted for their 500 progenies. GEBVs for all progeny of a given cross combination were generated using a genomic prediction model with the progeny as the validation set, and the parental lines, along with other inbred lines, used as the TS. Four genetic evaluation methods were used in this study for the calculation of progeny GEBVs: ridge regression BLUP (RR-BLUP) (Whittaker et al., 2000), Bayes B (BayesB) (Meuwissen et al., 2001), Bayesian ridge regression (BayesRR) (Perez et al., 2010), and extended genomic BLUP (EGBLUP) (Su et al., 2012). Both BayesB and RR-BLUP have been researched and utilized in multiple studies of various plant species (Whittaker et al., 2000; Meuwissen et al., 2001; Lorenz et al., 2011; Heslot et al., 2012; Wang et al., 2018). BayesRR relies on a shrinkage of marker effects similar to that in RR-BLUP which has been used successfully in prior research of soybean grain yield genomic selection (Perez et al., 2010; Duhnen et al., 2017). For both Bayes models (BayesB and BayesRR) 1,500 iterations were generated for each validation with a burn in of 500. EGBLUP resembles genomic BLUP but has an additional matrix which estimates additive by additive epistatic effects. This model was originally developed for animal breeding, but was found success in previous studies on soybean genomic selection (Su et al., 2012). The underlying assumptions of each model determine its predictive ability for a given trait, with the expectation that the model whose assumptions most closely match the genetic mechanisms controlling yield would lead to the highest prediction accuracies. The RR-BLUP model was fitted using the rrBLUP package (Endelman, 2011), BayesB and BayesRR were fitted using the bWGR package (Xavier et al., 2019; R Core Team, 2020), and EGBLUP was done using the EMMREML R package (Akdemir and Godfrey, 2015). Following the generation of GEBVs *via* genomic prediction, each cross’ value is assessed. The value of a cross consists of two components, the mean value of the cross’ progeny and the genetic variance among progeny (Mohammadi et al., 2015; Lehermeier et al., 2017). These two values are used to calculate a UC for a given cross:


$$UC_{m}=\mu +i\sigma_{g}$$

In which $UC_{m}$ is the cross value of a given cross m; μ is the mean phenotypic value of cross m’s progeny; i is selection intensity; and σ_g_ is the genetic standard deviation among cross progeny of m (Mohammadi et al., 2015). $V_{G}$ can be divided into three components, $V_{A}$ , $V_{D}$ , and $V_{I}$ , referring to genetic variance caused by additive, dominant, or epistatic effects. As $V_{D}$ is not present in RILs, $V_{I}$ is expected to be minimal and σ_g_ is equivalent to the standard deviation of GEBVs predicted *via* genomic prediction (Zhong and Jannink, 2007; Neyhart and Smith, 2019). Therefore, the UC can be calculated as the mean value among the top i proportion of GEBVs. For example, with a selection intensity of i = 0.1, the UC of a cross is equivalent to mean GEBVs of the top 10% of predicted GEBVs. Cross combinations were analyzed using a UC with a selection intensity of 0, 0.1, and 0.2 termed mean value (MV), $UC_{0.1}$ , and $UC_{0.2}$ , respectively (Zhong and Jannink, 2007; Bernardo, 2014; Mohammadi et al., 2015; Lehermeier et al., 2017).

#### 2.4.3 Optimal cross selection method development

As detailed above, there were multiple parameters which could be varied to create distinct OCS models that may differ in predictive ability. Within this experiment, 12 distinct OCS methods were tested to predict the values of crosses. These differed in the genomic prediction models (BayesB, BayesRR, RR-BLUP, and EGBLUP) that were used to predict genetic values as well as the UCs (MV, $UC_{0.1}$ , and $UC_{0.01}$ ) used to assess cross values. The qtl R package was used universally to generate inbred progeny genotypes for crosses. All code was executed in an R environment, utilizing dependencies and functions from the packages described above for their associated processes. The evaluation method of MV using the RR-BLUP model for marker effects was based on Mohammadi et al. (2015) in the PopVar model. All other methods used in this study were novel.

Following the development of multiple OCS methods, a key question was what conditions would affect their predictive accuracies. Prior research in genomic selection has indicated that TS formation can have a significant impact on predictive ability (Atanda et al., 2021; Zhu et al., 2021). Validation would then be focused on the conditions that OCS would be applied in a soybean breeding program, with a TS consisting of genetically disparate, elite breeding lines.

### 2.5 Training set composition & validation

Three TS compositions were utilized in validation, each with differing levels of genetic relatedness to the cross combinations predicted. The first composition, full training set (FTS), consisted of the full TS using all 702 lines, including the 35 parental lines used for validation. The second TS, without full siblings (WFS), removed all lines from the TS which were direct progeny of the crosses to be predicted, but still maintained all parental lines. The removal of full siblings from the TS is most similar to a breeding program’s TS, where a cross combination is only used once and is not repeated in later years. An additional TS composition was used to assess if any change in accuracy between FTS and WFS was due to the loss of relatedness between the TS and validation set or only due to a reduced TS size. The third composition, termed reduced training set (RTS), removed as many lines from the TS as WFS. In RTS all materials removed were randomly chosen lines that were not direct progeny of the predicted crosses. Therefore, the relatedness of the TS to the predicted crosses would be minimally impacted and WFS and RTS would have an equivalent number of plant materials. Additionally, predictions generated by OCS when utilizing the SoySNP6k marker set were compared to those generated when utilizing the SoySNP3k SNP marker set.

The validation for OCS consisted of predicting the value of 42 unique cross combinations made within the UGA Soybean Breeding Program. All crosses had at least 16 progenies evaluated in multi-location yield trials between 2015 and 2019 (**supplemental table 1**). The value of each cross was predicted using 12 combinations of four genomic prediction models (BayesB, BayesRR, RR-BLUP, & EGBLUP) and three assessment methods (MV, UC_0.1_, UC_0.2_). Accuracy was defined as the Spearman correlation between the values predicted for a given set of cross and the observed value of said set. Spearman correlation provides a comparison of rankings, making it an effective method to evaluate a OCS, which generates values that are used to rank and select crosses. The observed value of the crosses was the mean yield BLUP value of all progeny that were tested in AYTs for said cross.

Validation was carried out independently six times, for each combination of TS composition (FTS, WFS, & RTS) and marker sets (SoySNP6k & SoySNP3k). The validation process was identical for all six combinations. Validation for each marker set and TS consisted of 50 validation tests. In each test all 12 OCS evaluation methods predicted the progeny values of a random sample of 40 crosses from the 42 possible crosses. After progeny generation each genomic prediction method (BayesB, BayesRR, RR-BLUP, & EGBLUP) was utilized to determine the GEBVs of said progeny. Following this, OCS then assessed the value of each cross using all three UCs (MV, $UC_{0.1}$ , and $UC_{0.2}$ ). The prediction accuracy was recorded for each combination of genomic prediction model and analysis method.

## 3 Results

### 3.1 Heritability and genetic analysis

The heritability calculated from the 2015 to 2019 multi-location yield trials was highly variable and heavily impacted by genotype × environment interactions. A majority of trials during this time period consisted of two or more environments. In those yield tests at the multiple environments, the variance components of the genotype by environment interactions exceeded those of the genotypes in almost all instances. *H^2^* ranged from 0.09 to 0.74 on an entry mean basis with a mean heritability of 0.51 across all tests. Detailed information on each yield test and their heritability can be found in **supplemental file S2**.

**Figure 1:**
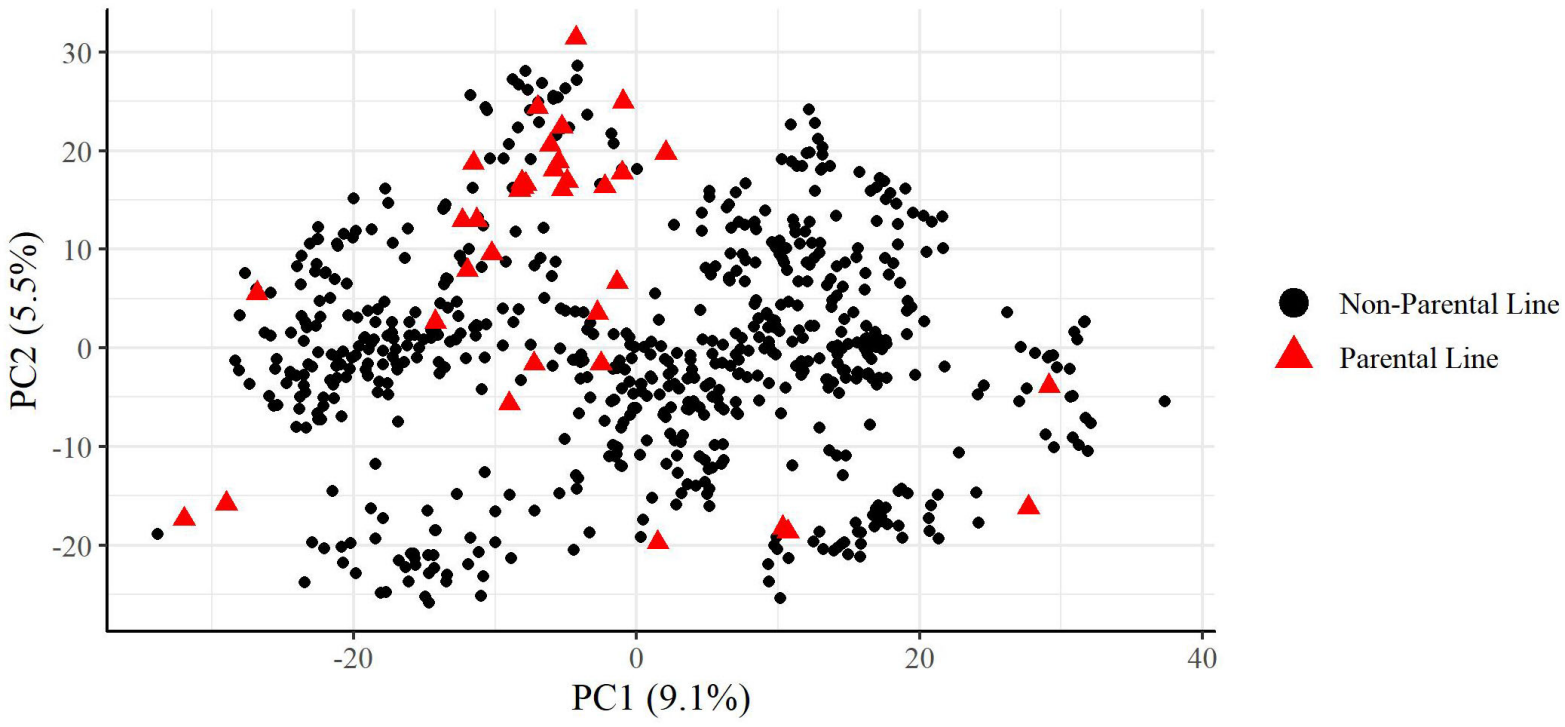
Principal components analysis of training set used in this study. Percentage values on each axis indicate percent of variation explained by that principal component. Parental lines are indicated as red triangles.

A principal component analysis of the plant materials indicated that the parental lines selected well represented the genetic variation found in the plant materials used in this study, with parental lines spreading across the full range of the first and second principal components which were plotted in **Figure 1**. Additionally, the plant materials did not show significant population structure which would interfere with analysis.

### 3.2 Comparison of genomic prediction models and analysis methods

Between all the parameters varied in this study, there were a total of 72 validation trials, each consisting of 50 replicates. These trials varied in terms of genomic evaluation model used, UC selected, TS composition, and marker sets. Variations in these parameters led to multiple statistically significant patterns and changes in prediction accuracy.

#### 3.2.1 Prediction using SoySNP6k marker set

The first 32 validation trials carried out in this study utilized the SoySNP6k marker set. The results of these 32 validation trials were compared using a Tukey HSD to determine statistically significant differences. In a comparison of models, EGBLUP was found to have a statistically higher predictive accuracy than all other models in all TS when using any UC (P< 0.001 in all comparisons). Variation in the UC only led to a statistical difference when using EGBLUP with the WFS TS. In this scenario MV had a statistically higher accuracy than UC_0.1_. EGBLUP had a prediction accuracy of 0.56 and 0.45 for FTS and RTS, respectively, and of 0.35 when using MV in WFS. The prediction accuracy of other three models depended upon the TS. Using the FTS and RTS TS there was not a statistical difference in the prediction accuracies of the three non-EGBLUP models at 0.37 for FTS and 0.33 for RTS. Under WFS validation, BayesB’s prediction accuracy, 0.26, was statistically below that of BayesRR, RR-BLUP, and EGBLUP (**Table 1**).

**Table 1:** Tukey honestly significant difference analysis of cross selection validation using a SoySNP6k marker set.

Usefulness Criterion	Cross Prediction Model	Full Training Set	Reduced Training Set	Without Full Siblings
UC_0.1_ ^a^	BayesB	0.37 | cd	0.32 | fghi	0.26 | j
	BayesRR	0.38 | c	0.33 | fg	0.29 | i
	EGBLUP	0.55 | a	0.46 | b	0.34 | def
	RR-BLUP	0.37 | cd	0.33 | f	0.29 | i
UC_0.2_ ^b^	BayesB	0.37 | cd	0.32 | fghi	0.26 | j
	BayesRR	0.37 | cd	0.33 | fg	0.29 | i
	EGBLUP	0.57 | a	0.47 | b	0.36 | cde
	RR-BLUP	0.37 | cd	0.33 | f	0.29 | hi
MV^c^	BayesB	0.38 | c	0.32 | fgh	0.26 | j
	BayesRR	0.38 | c	0.33 | f	0.29 | i
	EGBLUP	0.56 | a	0.47 | b	0.4 | c
	RR-BLUP	0.37 | cd	0.33 | ef	0.3 | ghi

a UC_0.1_ = Usefulness criterion with I = 0.1.

b UC_0.2_ = Usefulness criterion with i = 0.2.

c MV = Mean value, usefulness criterion with i = 0.

Note: Each test underwent 50 replications, with the mean value of those 50 replications shown and test followed by a different letter are significantly different based on Tukey HSD (α = 0.05).

**Figure 2:**
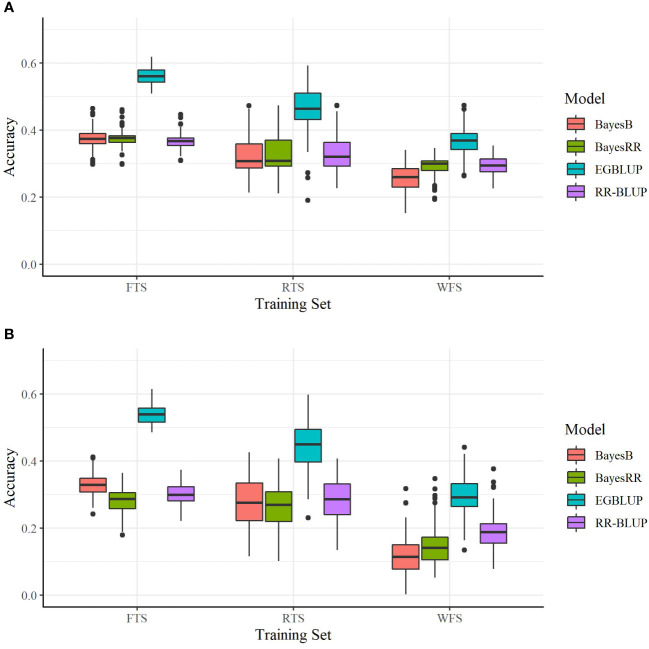
Prediction accuracies of four models utilizing two marker sets. **(A)** SoySNP6k SNP marker set and **(B)** SoySNP3k SNP marker set. Tested with the full training set (FTS), a training set of reduced size (RTS), or with all direct progeny of the predicted crosses removed (WFS). Each bar represents 150 validation trials.

The relationship between the crosses predicted and TS had an additional, statistically significant impact on prediction accuracy. For all OCS evaluation methods using the SoySNP6k marker set, there was a statistically significant increase (P< 0.001) in prediction accuracy when using RTS over WFS and FTS over RTS (**Figure 2A**). Therefore, predictive accuracy was reduced both by a reduction in TS size, as observed in the FTS to RTS comparison, and a reduction in relatedness, as observed in the RTS to WFS comparison.

#### 3.2.2 Prediction using SoySNP3k marker set

Overall, the results of OCS using the SoySNP3k marker set had similar patterns to those observed at higher marker densities. EGBLUP provided the prediction accuracies statistically higher than those of any of the other genomic evaluation models tested. Additionally, UC only had an impact on prediction accuracy for WFS using EGBLUP, leading to a statistically greater prediction accuracy in MV than in UC_0.1_ and UC_0.2_. EGBLUP had a prediction accuracy of 0.54 in FTS, 0.45 for RTS, and 0.35 using MV under WFS. The performance of the other genomic evaluation models relative to one another was dependent on the TS. Under FTS, BayesB had statistically higher mean prediction accuracy with a mean value of 0.33 than BayesRR, which had a mean prediction accuracy of 0.29. RR-BLUP’s results under FTS were statistically similar to those of BayesRR and BayesB with a mean predictive accuracy of 0.37. With the RTS, BayesB, BayesRR, and RR-BLUP had statistically similar results with a mean value of 0.27. In the WFS TS RR-BLUP’s prediction accuracy, with a mean value of 0.18, was statistically above that of BayesB, 0.12. BayesRR’s results in WFS were statistically similar to those of BayesB and RR-BLUP with a mean value of 0.14 (**Table 2**).

**Table 2:** Tukey honestly significant difference analysis of cross selection validation using a SoySNP3k SNP marker set.

Usefulness Criterion	Cross Prediction Model	Full Training Set	Reduced Training Set	Without Full Siblings
UC_0.1_ ^a^	BayesB	0.34 | cd	0.29 | fghi	0.11 | m
	BayesRR	0.29 | fghi	0.27 | ghi	0.13 | klm
	EGBLUP	0.53 | a	0.45 | b	0.25 | i
	RR-BLUP	0.3 | defg	0.29 | fghi	0.17 | jk
UC_0.2_ ^b^	BayesB	0.33 | cde	0.29 | fghi	0.12 | m
	BayesRR	0.29 | fghi	0.27 | ghi	0.14 | klm
	EGBLUP	0.54 | a	0.46 | b	0.28 | fghi
	RR-BLUP	0.3 | efgh	0.28 | fghi	0.18 | j
MV^c^	BayesB	0.32 | cdef	0.26 | hi	0.13 | lm
	BayesRR	0.28 | ghi	0.25 | i	0.16 | jkl
	EGBLUP	0.54 | a	0.43 | b	0.35 | c
	RR-BLUP	0.3 | defg	0.27 | ghi	0.2 | j

a UC_0.1_ = Usefulness criterion with i = 0.1.

b UC0.2 = Usefulness criterion with i = 0.02.

c MV = Mean value, usefulness criterion with i = 0.

Mote: Each test underwent 50 replications, with the mean value of those 50 replications shown and test followed by a different letter are significantly different based on Tukey HSD (α = 0.05).

As when using the SoySNP6k marker set, a reduction in TS relatedness to the crosses predicted led to lowered prediction accuracies at lower marker densities. For both EGBLUP and BayesB going from the FTS to RTS or RTS to WFS led to a statistically significant reduction in prediction accuracy. For RR-BLUP and BayesRR there was not a statistical difference between results from the FTS and RTS TS (**Table 2**). There was still a significant difference between RTS and WFS for those two models (**Figure 2B**).

#### 3.2.3 Effects of marker density

Within this study two SNP marker sets (SoySNP6k and SoySNP3k) were used. Overall, there was a statistically significant increase in accuracy for all OCS methods when using the higher marker densities (**Figures 2A, B**). In paired t tests between OCS methods using the 6k and 3k SNP marker sets, accuracy was statistically lower when using the 3k SNP markers for all OCS methods at a 0.05 confidence level with the exception of UC_0.1_ EGBLUP and UC_0.2_ EGBLUP using the RTS. On average, prediction accuracy was reduced by 0.06, 0.04, and 0.12 within the FTS, RTS, and WFS TS, respectively (**Table 3**). Though all TS compositions were affected, the difference in magnitude suggests that less related TS (WFS) are more strongly affected than those with higher relatedness to the crosses predicted. Between both marker sets, the greatest prediction accuracies were found when using the SoySNP6k marker set, with EGBLUP giving the highest prediction accuracies of the models tested in this study. In FTS the greatest prediction accuracy occurred with UC_0.2_ EGBLUP with a prediction accuracy of 0.57. MV EGBLUP was the best model tested in RTS and WFS with accuracies of 0.47 and 0.40, respectively.

**Table 3:** Comparison of prediction accuracy between SoySNP6k and SoySNP3k SNP marker sets.

Cross Prediction Model		Full Training Set		Reduced Training Set		Without Full Siblings		Average Difference
UC_0.1_ ^a^	BayesB	>0.01	0.03	0.02	0.03	>0.01	0.15	0.07
	BayesRR	>0.01	0.09	>0.01	0.06	>0.01	0.16	0.1
	EGBLUP	>0.01	0.02	0.27	0.01	>0.01	0.09	0.04
	RR-BLUP	>0.01	0.07	>0.01	0.04	>0.01	0.12	0.08
UC_0.2_ ^b^	BayesB	>0.01	0.04	0.01	0.04	>0.01	0.14	0.07
	BayesRR	>0.01	0.09	>0.01	0.06	>0.01	0.15	0.1
	EGBLUP	>0.01	0.03	0.33	0.01	>0.01	0.08	0.04
	RR-BLUP	>0.01	0.07	>0.01	0.05	>0.01	0.11	0.08
MV	BayesB	>0.01	0.06	>0.01	0.06	>0.01	0.13	0.08
	BayesRR	>0.01	0.1	>0.01	0.07	>0.01	0.13	0.10
	EGBLUP	>0.01	0.02	0.03	0.03	>0.01	0.04	0.03
	RR-BLUP	>0.01	0.07	>0.01	0.06	>0.01	0.09	0.07
Average Difference			0.06		0.04		0.12	

a UC_0.1_ = Usefulness criterion with i = 0.1.

b UC_0.2_ = Usefulness criterion with i = 0.2.

c MV = Mean value, usefulness criterion with i = 0.

Note: The first (center) value indicates the p value for the associated test, the second value (right) indicates the estimated difference in mean prediction accuracies. A positive value indicates that the predictions with the SoySNP6k marker set are more accurate.

## 4 Discussion

Yield is the primary agronomic trait of soybean with a complex, quantitative genetic architecture and a low heritability relative to soybean’s seed composition traits (Bernardo, 2002; Diers et al., 2018; Đorđević et al., 2019). In this study multiple OCS methods were developed and validated empirically within a soybean breeding program. While prior studies have tested cross prediction methods, these have primarily been validated *in silico* or with specialized populations and TS (Lehermeier et al., 2017; Neyhart and Smith, 2019).

### 4.1 Parameters for OCS

Within validation there were significant differences in predictive accuracies among the OCS methods developed for this study. Of the methods assessed, those using EGBLUP were preferable, providing the highest prediction accuracy. MV EGBLUP in the WFS TS, which is the closest to breeding program conditions, achieved an accuracy of 0.4 using the SoySNP6k marker set, the highest observed for WFS. EGBLUP’s unique factor is its inclusion of an additive by additive epistatic effect matrix, as it otherwise resembles genomic BLUP or RR-BLUP. The performance of EGBLUP in this study suggests that epistatic, additive by additive effects are present in elite soybean germplasm. The findings of this research indicate the incorporation of these effects into OCS by usage of the EGBLUP genomic evaluation is key to the cross assessment and prediction.

A prediction accuracy of 0.4 is comparable to prediction accuracies reported for cross population genomic selection of soybean yield. Genomic selection accuracies for yield in winter wheat and soybean are reported with values typically between 0.5 and 0.7, (Norman et al., 2018; Jarquin et al., 2019; Zhu et al., 2021). However, a majority of these results come from genomic selection in which the plant materials are all members of the same breeding populations or specially formed bi-parental populations. In these studies, the TS and validation set are more closely related than they would be in a breeding program and may even be full siblings, leading to an increased accuracy of genomic selection (Michel et al., 2016; Sun et al., 2019; Zhu et al., 2021). However, this is not truly applicable to the plant breeding process. In experiments explicitly investigating genomic prediction for yield across breeding cycles the range of reported accuracies is much lower, 0.2 to 0.4, which the WFS prediction accuracies found in this study equal and exceed (Michel et al., 2016; Atanda et al., 2021).

These latter validation scenarios are closer to those which would be experienced within a breeding program. New inbred lines within a breeding program, which would be the targets of genomic selection, have pedigrees distinct from those of prior varieties which would be included in the TS. This set of conditions closely follows the assumptions of the WFS TS in this study, in which there are no directly related lines to the generated progeny which must be evaluated by OCS. As the highest prediction accuracy under WFS was 0.4, OCS achieves prediction accuracies within the same range as genomic selection in cross environment predictions for yield.

#### 4.1.1 Genetic relatedness affects predictive ability

Within this study there was a loss of prediction accuracy as relatedness was reduced between the TS and the crosses predicted. This occurred to a statistically significant degree, universally when using the SoySNP6k marker set, and between WFS and the other TS compositions when using the SoySNP3k marker set. The impact of TS relatedness and size on prediction accuracy is supported by prior genomic selection and TS optimization research (Lorenz and Smith, 2015; Stewart-Brown et al., 2019; Zhu et al., 2021). Our results further indicated that the relatedness of material within the TS is more important than the size of said TS, as results using the RTS were statistically superior to those from the same OCS methods using the WFS TS. These findings suggest that TS formation and optimization for OCS can use the same techniques as those deployed in TS optimization for genomic selection (Lorenz and Smith, 2015; Zhao et al., 2015; Đorđević et al., 2019; Atanda et al., 2021).

#### 4.1.2 Impacts of marker density on predictive ability

An additional factor impacting prediction accuracy was the marker density used. The results indicated the SoySNP6k marker set led to statistically greater prediction accuracies than the SoySNP3k marker set in almost all OCS methods and TS compositions used. Though there was a decrease in prediction accuracy for all TS when using the SoySNP3k marker set rather than the SoySNP3k marker set, the loss of accuracy was greatest in WFS, with a decrease of 0.12 on average, double that of RTS (0.04) and FTS (0.06) (**Table 3**). Prior research has shown that a reduction in marker density can lead to reduction in genomic prediction accuracy to yield (Ma et al., 2016; Đorđević et al., 2019; Stewart-Brown et al., 2019). However, within soybean the necessary marker density to reach a plateau in prediction accuracy, where adding additional markers has no impact, is considered to be low and the ~2000 polymorphic markers within the 3k SNP marker are expected to meet said plateau (Wang et al., 2018; Đorđević et al., 2019; Stewart-Brown et al., 2019).

These results suggest that genetic coverage is more important to successful OCS than it is for genomic selection. Considering that OCS must generate and predict progeny genotypes and values, the increased marker density will lead to more accurate generation of progeny genotypes. With a reduced marker density, the linkage between markers and QTL is minimized, therefore progeny generated using lower marker densities less accurately represent the progeny which could result from a cross. This makes the subsequent prediction of progeny values and cross values more difficult, making SoySNP6k preferable for OCS over SoySNP3k (Wang et al., 2018; Đorđević et al., 2019; Stewart-Brown et al., 2019).

### 4.2 Deployment of OCS in an applied breeding programs

Within this study the highest prediction accuracies were observed when using the mean value EGBLUP method with the SoySNP6k marker set, 0.40 (WFS) and 0.56 (FTS). Therefore, the MV EGBLUP OCS model using the SoySNP6k marker set is recommended for the prediction of yield cross values in soybean. Limited studies have validated OCS in an empirical manner, and none have done so for soybean yield (Mohammadi et al., 2015; Neyhart and Smith, 2019). Neyhart and Smith (2019) empirically validated an OCS model, developing 27 unique barley crosses until they could be evaluated for plant height, fusarium head blight (FHB) severity, and heading date. In predicting these traits, their OCS model achieved a prediction accuracy of 0.53 (height), 0.46 (FHB), and 0.62 (heading). Of these traits, FHB severity was noted to have the lowest broad-sense heritability of 0.46, roughly equivalent to the mean heritability observed in this study’s yield trials. Within Neyhart and Smith (2019) only 12 families were predicted to validate FHB severity as compared to the 42 families used within this study. With a limited sample size of 12 crosses to predict it is possible the predictive ability observed could be an outlier, a possibility acknowledged by the researchers, and could have a lower true mean predictive accuracy when used to predict a larger number of crosses.

The successful use of the OCS method recommended here depends on a number of factors and is beholden to some limitations. The predictive accuracy of OCS is constrained by the phenotypic and genotypic data used in the TS. The phenotypic data used in the TS and for validation was gathered in the same geographic area in the southern United States. The prediction accuracy of OCS would be reduced if it were used to predict the performance of crosses in a different geographic region than the TS was evaluated in. Furthermore, OCS requires an efficient genotyping pipeline to operate, as genotypic and phenotypic data for the parental lines must be available prior to planting. This could cause issues dependent on the genotyping resources and throughput available to a breeding program. Computational resources are additionally essential to OCS as the prediction and evaluation of crosses can be computationally demanding. This is especially true for methods involving EGBLUP which took ~12x longer to finish validation than the other genomic evaluation models on a high-performance desktop computer. Given sufficient access to computational resources, the time to execute OCS in a breeding program would be less than 24 hours.

This study presents multiple future research direction and utilizations. First, OCS could be used to select for multiple traits at once, with crosses selected based on an index of weighted traits values such as yield, seed composition, disease, or nematode resistance. This method could identify populations with progeny that have both desirable yield and seed composition traits, which can be antagonistic. Additionally, QTLs with known effects could be included as weighted covariates in progeny evaluation for more heritable traits, such as maturity, disease, and nematode resistance (Yao et al., 2018; Neyhart et al., 2019). For pest and disease resistance, OCS could allow breeders to determine how likely it is for progeny to possess the resistance QTL from both parents for multiple traits. This would be especially helpful for resistance QTL which are in tight linkage with one another. For soybean specifically, prediction of maturity using known maturity genes/QTLs could allow breeders to make crosses between maturity groups. OCS in this instance would provide a range of maturities for the progeny of a given cross, allowing breeders to identify which regions the progeny of such a cross would be suitable for. This would increase the variety of germplasm that breeding programs have access to beyond the typically narrow range of maturity groups (Zimmer et al., 2021). Multiple studies have identified maturity QTLs which could be utilized in OCS for maturity prediction (Langewisch et al., 2017; Zimmer et al., 2021).

## 5 Conclusion

Determination of cross combinations is one of essential steps in soybean breeding. OCS enables parental selection decisions based on genetic evaluation and simulation prior to crossing, which could lead to higher efficiency in the breeding pipeline and greater genetic gains. OCS considers complementation between parental genotypes and allows a breeder to evaluate the distribution of progeny values from a cross combination. Result indicated that MV EGBLUP using the SoySNP6k marker set is the most accurate OCS method of those tested, with a prediction accuracy of 0.4 when using a breeding program TS. OCS is highly impacted by the relatedness of the TS to the cross combinations predicted, marker density, and the genomic evaluation model used. The predictive methodologies generated in this study can be utilized by soybean breeding programs as well as breeding programs for other crop species leading to improved rates of genetic gain.

## Data availability statement

The raw data supporting the conclusions of this article will be made available by the authors, without undue reservation.

## Author contributions

MM conducted genetic experiments, analyzed data, interpreted the results and drafted the manuscript. ZL conceptualized the project, provided oversight of the experiments, interpreted the results, and edited the manuscript. QS carried out the genotyping of all experimental lines, analyzed data, and interpreted the results. BF conducted the field tests and collected agronomic trait data. All authors contributed to the article and approved the submitted version.
