# Supplementary material for: Genomic prediction of optimal cross combinations to accelerate genetic improvement of soybean (Glycine max)
Source: Front Plant Sci. 2023 May 10;14:1171135. doi: 10.3389/fpls.2023.1171135 (PMC10206060; doi:10.3389/fpls.2023.1171135)
Supplement: Supplementary file 2 [file Table_1.docx]

**Table S1.** Pedigrees and number of progeny derived that were used in the validation

| \| Cross combination \| Number of RILs tested \| \| --- \| --- \| \| G10PR-56444R2 × G10PR-224R2 \| 16 \| \| N05-7432 × G10PR-56248R2 \| 16 \| \| G09PR-54329R2 × N02-7084 \| 17 \| \| G11-1162R2 × G10PR-56444R2 \| 17 \| \| G08PR-394 × G10PR-56389R2 \| 18 \| \| NCC04-619 × G09PR-54457R2 \| 18 \| \| R04-342 × G09PR-54457R2 \| 19 \| \| N08-145 × G10PR-56248R2 \| 20 \| \| NMS4-1-77 × G10PR-56248R2 \| 21 \| \| NMS4-1-77 × G10PR-56466R2 \| 21 \| \| N06-10237 × G10PR-56248R2 \| 22 \| \| NCC06-899 × G10PR-56248R2 \| 22 \| \| G10PR-224R2 × G11-1162R2 \| 23 \| \| N08-521 × G10PR-56466R2 \| 23 \| \| NCC06-899 × G10PR-56389R2 \| 23 \| \| G10PR-86R2 × G10PR-56330R2 \| 25 \| \| G11-1162R2 × G11-1762R2 \| 26 \| \| G10PR-56444R2 × G11PR-407R2 \| 27 \| \| N06-10237 × G10PR-56466R2 \| 29 \| \| N08-391 × G10PR-56466R2 \| 30 \| \| G11-1162R2 × G11-1397R2 \| 32 \| \| N05-7432 × G09PR-54329R2 \| 32 \| \| N05-7432 × G10PR-56466R2 \| 32 \| \| R07-10322 × G10PR-56389R2 \| 33 \| \| G09PR-58 × G09PR-54329R2 \| 34 \| \| G10PR-56248R2 × G10PR-56389R2 \| 35 \| \| G08PR-394 × G09PR-54329R2 \| 36 \| \| N05-7462 × G09PR-54329R2 \| 36 \| \| NCC06-1090 × G10PR-56248R2 \| 36 \| \| N08-391 × G10PR-56248R2 \| 38 \| \| N08-521 × G10PR-56248R2 \| 38 \| \| G00-3880R2 × Benning EMGH \| 42 \| \| G00-3213R2 × Benning EMGH \| 43 \| \| N05-7432 × G09PR-54457R2 \| 43 \| \| R04-522 × G10PR-56248R2 \| 44 \| \| G93-2225 × G09PR-54329R2 \| 46 \| \| N07-14182 × G10PR-56248R2 \| 46 \| \| NCC06-1090 × G10PR-56389R2 \| 53 \| \| G10PR-86R2 × G10PR-224R2 \| 55 \| \| G08PR-394 × G10PR-56248R2 \| 61 \| \| NCC07-8138 × G10PR-56248R2 \| 64 \| \| NCC07-8138 × G10PR-56389R2 \| 67 \| \| Average \| 33 \| |
| --- | --- | --- | --- | --- | --- | --- | --- | --- | --- | --- | --- | --- | --- | --- | --- | --- | --- | --- | --- | --- | --- | --- | --- | --- | --- | --- | --- | --- | --- | --- | --- | --- | --- | --- | --- | --- | --- | --- | --- | --- | --- | --- | --- | --- | --- | --- | --- | --- | --- | --- | --- | --- | --- | --- | --- | --- | --- | --- | --- | --- | --- | --- | --- | --- | --- | --- | --- | --- | --- | --- | --- | --- | --- | --- | --- | --- | --- | --- | --- | --- | --- | --- | --- | --- | --- | --- | --- | --- |
|  |
|  |
|  |

**Table S2**. Heritability of different advanced yield test (AYT) sets across years

| Year | AYT1 | AYT2 | AYT3 | AYT4 | AYT5 |
| --- | --- | --- | --- | --- | --- |
| 2015 | 0.35 | 0.09 | 0.5 | 0.63 | N/A |
| 2016 | 0.21 | 0.52 | 0.51 | 0.65 | 0.65 |
| 2017 | 0.67 | 0.58 | 0.38 | 0.63 | 0.63 |
| 2018 | 0.67 | 0.74 | 0.46 | 0.66 | N/A |
| 2019 | 0.45 | 0.31 | 0.36 | 0.63 | N/A |
